# A Novel Apparatus for Simultaneous Laser-Light-Sheet Optical Particle Counting and Video Recording in the Same Measurement Chamber at High Temperature

**DOI:** 10.3390/s22041363

**Published:** 2022-02-10

**Authors:** Julian Zoller, Amin Zargaran, Kamil Braschke, Jörg Meyer, Uwe Janoske, Achim Dittler

**Affiliations:** 1 Karlsruhe Institute of Technology, Institute of Mechanical Engineering and Mechanics, Straße am Forum 8, 76131 Karlsruhe, Germany; joerg.meyer@kit.edu (J.M.); achim.dittler@kit.edu (A.D.); 2 School of Mechanical Engineering and Safety Engineering, Bergische Universität Wuppertal, Gaußstraße 20, 42119 Wuppertal, Germany; zargaran@uni-wuppertal.de (A.Z.); braschke@uni-wuppertal.de (K.B.); janoske@uni-wuppertal.de (U.J.)

**Keywords:** optical particle counter, high temperature, video recording, single fibre, detachment

## Abstract

A novel apparatus was developed, to investigate the detachment of particle structures consisting of soot and ash from a single fibre or a fibre array in hot gas flow. Key features of the novel apparatus are operation at high temperatures while two different measurement techniques are applied simultaneously in the same measurement chamber to observe particle structure detachment from a loaded fibre array. A heated inlet can heat the air stream at the position of the fibre array up to 470 °C, allowing detachment investigations at temperatures relevant for the operation of, e.g., soot particle filters. The first measurement technique integrated in the setup is video recording of the fibre array, which gives qualitative information on the rearrangement or detachment of particulate matter on the fibre. Because it is often difficult to distinguish rearrangement and detachment from pure visual observations, a second measurement technique is applied. This technique is a laser-light-sheet optical particle counter, which can detect detached particle structures and determine their size. The measurable size range is 257 to 1523 µm for glass spheres. This paper presents and discusses the novel apparatus, its calibration and first detachment measurement results.

## 1. Introduction

**Figure 1:**
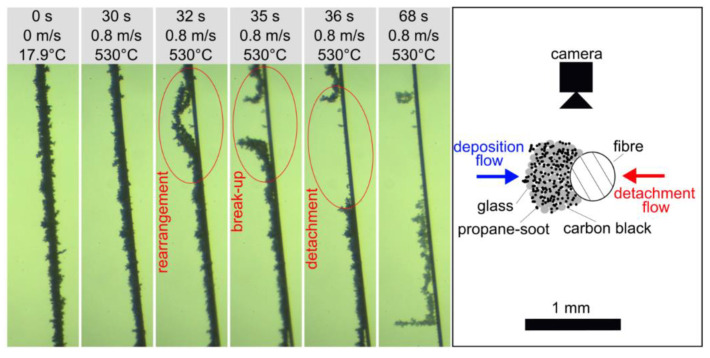
Detachment of particle structures consisting of propane-soot (volume median diameter $x_{50,3}=0.352\;$µm), carbon black ($x_{50,3}=$ 0.445 µm) and glass spheres ($x_{50,3}=6.8$ µm) from a single 40 µm steel fibre in hot air flow with an average flow velocity of 0.8 m/s at 530 °C. This experiment was made with the experimental setup described in [4].

Deposition of reactive and inert particles on filter media or technical equipment is observed in many applications such as the treatment of exhaust gas [1] or synthesis gas [2,3]. The collectors on which those deposits are formed are often cylindrical, for instance the cylindrical fibres in depth filters. Detachment of particulate deposits from the collector can occur due to flow forces. This detachment can be intended in a cleaning or regeneration process but can also be unintentional for example during particle separation in a fibrous depth filter. Reaction and disappearance of a particle structure component, for example the oxidation of soot, can change the deposited particle structure and thus the detachment [4]. Figure 1 shows the detachment of a particle structure consisting of propane soot (miniCast 6204C, Jing Ltd, Zollikofen, Switzerland), carbon black (Pow Carbon 280, Harold Scholz & Co. GmbH, Recklinghausen, Germany) and glass spheres (Spheriglass 5000CP00, Potters Industries LLC, Malvern, PA, USA) for illustration. The purpose of the novel apparatus presented in this article is to allow investigation of this detachment phenomenon and it is able to produce hot air jets, observe the particle structure on the fibre during the detachment experiments and detect detached agglomerates.

A former study [4] with propane–soot and glass spheres on a single filter fibre in cross flow showed that the detachment of deposited soot–glass agglomerates can be enhanced by the combustion of the soot, so that it occurs at lower average flow velocities of 0.5 m/s instead of 1.9 to 2.6 m/s. This study also showed an influence of the temperature on the detachment process even without reaction.

A drawback of the former study is that the detachment process was only investigated by video analysis in a simple open setup (analogous to the experiment in Figure 1), which leads to errors due to streaks, rearrangement of the particle structure and rotation of the fibre. Another drawback was the rather inaccurate and imprecise flow and temperature conditions during the experiments. The novel apparatus, presented in this paper, was developed in order to overcome these drawbacks. The apparatus includes a closed system with an improved inlet in the measurement chamber, a camera for video recording of the fibre and a laser-light-sheet optical particle counter (LLS-OPC). The improved inlet allows exposure of the fibres and particulate deposits on the fibres to a heated air flow. The video camera is used to observe the rearrangement processes on the fibre, but is not able to clearly detect detachment. The LLS-OPC allows the detection of the detached particulate structures and the determination of their size, but cannot measure the rearrangement of particulates on the fibres. 

A similar LLS-OPC was first presented by Wurster et al. [5,6] for the detection of oil drops entrained from filter media at ambient temperature and without simultaneous video recording. Wurster expanded a 1 W, 532 nm laser beam (MGL-FN-532, manufacturer PhotonTec Berlin GmbH, Berlin, Germany) with a Gaussian intensity distribution to a 400 mm light sheet using a cylindrical lens. Only the 80 mm middle section of the light sheet was used for particle counting because the light intensity variation is small in this section. This LLS was positioned directly downstream the filter media in order to observe the complete backside area of the filter media. The light scattered on oil droplets was detected by a photomultiplier (P30CW5, manufacturer Sens-Tech Ltd, Egham, UK) and hence oil drops in the size range of 170 to 2340 µm could be detected and their size determined.

There are several issues in making an optical particle counter temperature resistant. The heat radiation may disturb particle measurement, and hence the wavelength region of high photomultiplier sensitivity and laser radiation should be chosen far away from the region of heat radiation [7]. Additionally, an optical filter can reduce the influence of heat radiation on the photomultiplier signal. The optical components have to be protected against the temperature using appropriate cooling and distance [8].

The present study shows an advancement of the LLS-OPC presented by Wurster et al. With the new apparatus, videos of the observed object can be captured simultaneously in the same measurement chamber as the LLS at temperatures up to 470 °C. Particle agglomerates containing propane–soot and glass spheres are used in this study. The different light scattering properties of soot and glass spheres are discussed in Section 3.1.

## 2. Materials and Methods

### 2.1. The Novel Apparatus

**Figure 2:**
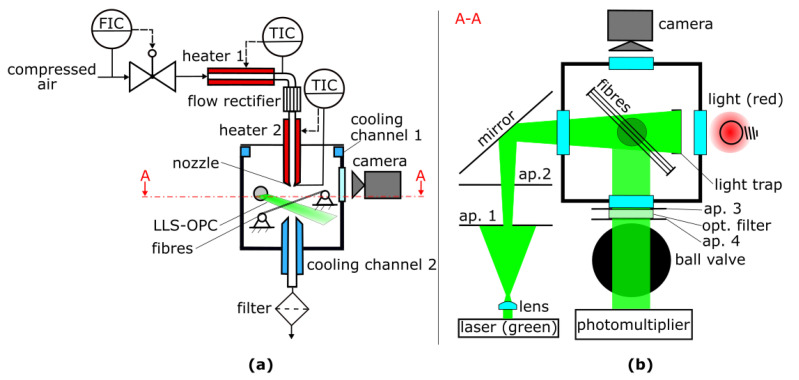
(**a**) Concept of the novel apparatus with the measurement chamber and the heated inlet; (**b**) Optical measurement system on the right side. The schematic diagram is not true to scale.

Soot particles and glass spheres, used as model ash, are deposited on parallel fibres using a combination of a dust disperser (SAG 410U, manufacturer Topas GmbH, Dresden, Germany) and a soot generator (miniCast 6204C, manufacturer Jing Ltd, Singapore) [9]. The particle-loaded fibre array is then positioned inside the novel apparatus shown schematically in Figure 2.

**Figure 3:**
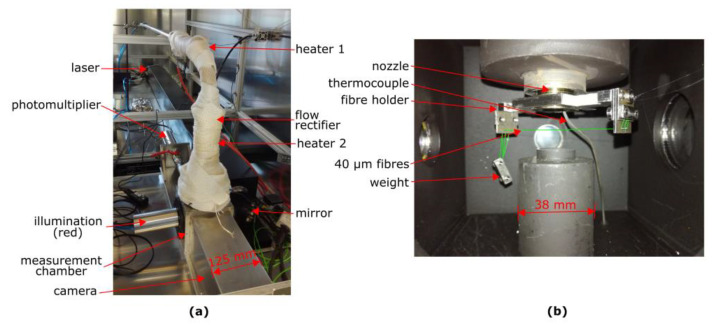
(**a**) Photograph of the novel apparatus; (**b**) Fibres mounted inside the measurement chamber. The fibres are marked with green lines in the right photograph.

Photographs of the novel apparatus and the fibres mounted in the inside of the measurement chamber are shown in Figure 3. The 40 µm steel fibres can be mounted in 25 different holes with 170 µm diameter at a centre-to-centre distance of 246 µm and tensioned using a weight fixed at one end of the fibres.

A defined mass flow (controlled by a MFC 1559A-100L-SV, manufacturer MKS Instruments Inc. Andover, MA, USA) of compressed air is heated in heater 1 (HotCoil 230 V/1600 W, max. 750 °C, 250 mm long, manufacturer Friedr. Freek GmbH, Menden, Germany) to a temperature T1. Heater 1 is positioned horizontally to reduce flow instabilities due to convectional flow [10,11]. The air is redirected downwards by flowing through a pipe elbow and a flow rectifier (Cordierit honeycombs, 200 cpsi, 59 mm long, 1.5 × 1.5 mm^2^ channels, manufacturer Fraunhofer IKTS). The flow should impinge on the fibre array vertically from above because then the detached particle structures follow gravity and sediment through the LLS. In addition, a horizontal free jet of hot air would have no homogeneous temperature and velocity profile because it bends upward. Downstream of the flow rectifier is a second heater (HotCoil 230 V/1400 W, max. 750 °C, 200 mm long, manufacturer Friedr. Freek GmbH, Menden, Germany), which heats the flow tube to a temperature T2 < T1. The reason for the slightly lower temperature of heater 2 is that lower temperatures at the tubing walls increase the density of air near the walls, which increases gravitational forces in the downward direction in this region and hence should lead to a plug flow shaped velocity profile. If the tubing walls of heater 2 would be hotter than those of heater 1, the reduced air density at the tubing walls together with the flow boundary layer can lead to convective flow in the opposite direction (to the top) and hence induce flow instabilities [11]. The heated air is directed at the fibre array as a free jet after flowing through a nozzle for further homogenisation of the flow and temperature profile (results regarding the flow and temperature profile are shown in Section 3.1).

The hot air causes oxidation of the soot particles in the particle structures, which were deposited on several parallel fibres. This oxidation, combined with drag force and gravity, can result in rearrangement and detachment of parts of the residual particle structure. Both effects can be observed in the novel system. The integration of the optical measurement system, consisting of the LLS-OPC for detection of detached agglomerates and the camera for observation of rearrangement of particle structures on the fibre array, is depicted on the right side of Figure 2.

An LLS-OPC is used to count and characterize detached particles with respect to their size/ volume. Therefore, the continuous 2 mm Ø beam of a 1 W laser with a Gaussian intensity profile (TEM00) and a wavelength of 532 nm (MGL-FN-532, manufacturer PhotonTec Berlin GmbH, Berlin, Germany) is expanded to a light sheet in horizontal direction using a cylindrical plano-convex lens (318322000, 61.31 mm focal length, manufacturer Qioptiq Photonics GmbH & Co. KG, Regen, Germany). Only the middle section of the generated laser-light-sheet is used for particle measurement because it has a nearly constant radiation intensity along the width of the light sheet. The 10 mm middle section is cut out of the 50 mm light sheet with aperture 1 (ap. 1). Aperture 2 (ap. 2) is wider than the light sheet and only reduces the amount of light scattered on aperture 1 which could reach the measurement chamber. The light sheet is directed inside the measurement chamber below the fibre array by a mirror and terminates in a light trap. The mirror can be used for adjustment of the light sheet position. The path length of the laser light from the lens to aperture 1 is 1694 mm and from aperture 1 to the middle of the measurement chamber is 847 mm. This long path is needed to expand the middle section of the laser light sheet to a width of 15.2 mm. Another lens with a shorter focal length and a different aperture could shorten this length, but then the width of the light sheet, and hence its radiation intensity would vary significantly below the fibre array, leading to measurement errors. Therefore, a relative long path length is chosen, for which the decrease in the LLS intensity below the fibre array is negligible. Particle structures, which detach from the fibre array and pass through the light sheet scatter light from the laser sheet. The scattered light is detected by a photomultiplier (P30CW5, manufacturer Sens-Tech Ltd, Surrey, UK), positioned perpendicular to the incident light beam and the flow direction. Between the photomultiplier and the measurement chamber, two apertures with 22 mm diameter are positioned to generate a defined observation area below the fibre array, and an optical filter (DT-Green, manufacturer Optics Balzers AG, Balzers, Liechtenstein) is installed, which hinders heat radiation and red light from the illumination for the camera observation from reaching the photomultiplier. The photomultiplier signal voltage is amplified by a factor of 4, filtered (low pass filter with cutoff frequency of 100 kHz) and captured by a PC-oscilloscope (PicoScope 4224, manufacturer Pico Technology Ltd., Cambridgeshire, UK) and the data are sent to a computer, where it is processed using LabVIEW (manufacturer, National Instruments Corporation, Austin, TX, USA). 

A camera is used for observation of the particle structures on the fibre array. The fibre array is illuminated by red light (LED-Modul 21 mm, red 625 nm, flood 28, IL-100, manufacturer StarLight Opto-Electronics GmbH & Co. KG, Nuremberg, Germany) and observed by a CMOS USB 3.0-camera (acA4024-29uc Basler ace, manufacturer Basler AG, Ahrensburg, Germany) equipped with a 6.5 × zoom with fine focus lens (1-6232, manufacturer Navitar Industries LLC, Singapore) and a 0.67 × adapter (1-6020, manufacturer Navitar Industries LLC, Singapore). The camera system is mounted on a three-point positioning system, which allows adjustment of its height and angle to the horizontal plane.

### 2.2. Method for Calibration and Characterization of the Novel Apparatus

The hot air jet is characterized with a prandtl-tube consisting of two tubes with a diameter of 1.1 mm each, one for total pressure and one for static pressure. The tubes are connected to a differential pressure measurement device (testo 521, manufacturer Testo SE & Co. KGaA, Baden-Württemberg, Germany). The prandtl-tube is equipped with a 0.25 mm thermocouple (type k, TC Mess- und Regeltechnik with testo 925 thermometer, manufacturer Testo SE & Co. KGaA, Baden-Württemberg, Germany). The prandtl-tube is fixed at the height of the fibres and can be positioned horizontally using an xy-stage.

The homogeneity of the LLS is examined by placing a 34 µm Trevira Monofil® fibre (manufacturer Trevira GmbH, Bobingen, Germany) perpendicular to the LLS at different positions within the LLS and measuring the voltage signal of the photomultiplier. The tested positions are the centre, the nearest position to the laser-light-sheet inlet in the chamber, the nearest position to the camera, the nearest position to the light trap and the nearest position to the photomultiplier inside the relevant measurement region below the nozzle opening.

Calibration of the laser light sheet is performed by measuring the signals of particles with a known size and composition. The size of the particles is described by the Feret diameter, which is determined by the analysis of microscopic images of particles in the according size fraction. Glass spheres and activated hard coal particles (Prestige Aktive Steinkohle, supplier Gert Strand AB, Hvidovre, Denmark) are used for calibration. The particles are picked with a tweezer and are dropped in the inlet of the measurement chamber. The measurements are performed at ambient temperature (22 °C) and elevated temperature (380 °C nozzle temperature) with and without additional illumination of the measurement chamber by the red light (for camera observation) and with and without three parallel mounted fibres in the measurement chamber for comparison. The photomultiplier amplification voltage is 356 V and the flow rate through the measurement chamber is 4 L/min.

### 2.3. Method for Investigation of Reactive-inert Particle Structure Detachment from a Fibre Array

In order to proof the function of the novel apparatus detachment experiments were made. Three parallel 40 µm steel fibres with a distance of 2.46 mm from each other were exposed first to an aerosol containing soot (352 nm volume median diameter, 5.3 × 10^7^ cm^−3^ number concentration) for 120 min. and second to an aerosol containing glass spheres (6.8 µm volume median diameter, 1.0 × 10^5^ cm^−3^ number concentration) for 7 min. at an average flow velocity of 52 cm/s in both cases. This sequential particle deposition showed to enhance the detachment of particulate structures especially at high temperatures. After particle deposition, the loaded fibre array is mounted in the new apparatus and exposed to a stepwise increasing flow velocity at a nozzle temperature of 380 °C and a tube temperature downstream heater 1 of 390 °C to observe the detachment and reaction on the fibre and detect detached particles. The first step at a velocity of 1 m/s is held for 5 minto achieve complete soot oxidation before the velocity is further increased in 30 s steps to observe detachment. The reason for this procedure is that sizing of detached agglomerates consisting solely of glass spheres is easier than sizing of agglomerates consisting of soot and glass spheres as discussed in Section 3.1.

## 3. Results and Discussion

### 3.1. Calibration and Characterization Results

**Figure 4:**
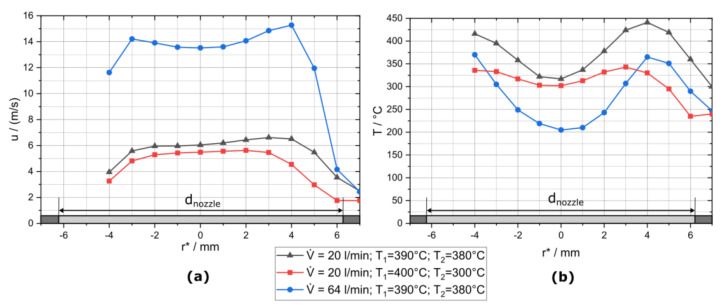
(**a**) Velocity and (**b**) temperature profile at the height of the fibres (10 mm distance from the nozzle outlet).

The temperature and velocity profiles at the height of the fibres (in 10 mm distance from the nozzle outlet) are shown in Figure 4. The nozzle diameter is 12.5 mm, and the radial coordinate $r^{*}$ is determined by placing the prandtl-tube at the edge of the 16 mm diameter outlet tube. The radial coordinate is zero at the nozzle axis, positive in one radial direction away from the nozzle axis and negative in the other direction. The measurable distance of the xy-stage is not sufficient to measure the complete distance in the negative $r^{*}$ direction, and there may be some misalignment between the real radial coordinate of the nozzle r and the radial coordinate $r^{*}$.

The left diagram in Figure 4 shows that the flow can be described as a plug flow. Flow velocity seem to decrease in the negative $r^{*}$ direction, but this may be caused by a shift in the differential-pressure measurement device with time, even without flow and a slight angle of the prandtl-tube to the nozzle axis. The temperature of the gas jet shown on the right side of Figure 4 can be higher than the tube temperature at the outlet of heater 1 ($T_{1}$) and the nozzle temperature ($T_{2}$) because the corresponding thermocouples are not positioned in the gas flow and are some distance apart from the heaters (in colder regions). The temperature is not constant over the radial coordinate of the hot air jet. The temperature in the middle of the air jet can be more than 100 °C lower than at the outside regions, which may be caused by heater 2, which heats the gas flow in the tube from the outside. This temperature difference can be decreased by increasing the tube temperature at the outlet of heater 1 to 400 °C and decreasing the nozzle temperature to 300 °C as shown by the red curve with rectangles in the right diagram of Figure 4. However, the nozzle temperature has to be higher than 300 °C to provide sufficient temperature for soot oxidation (about 350 °C). The temperature and velocity profiles are stable and do not show the flow instabilities reported for downward heated flows in the literature [10,11].

A correction of the measured temperature regarding heat radiation was not performed because Roberts et al. [12] reported an error for a sheathed thermocouple of 38.3 °C at a thermocouple reading of 591.8 °C, which is below 10% of the measured value. The error decreases for lower temperatures and is thus negligible in this study with temperatures up to 470 °C.

The homogeneity of the LLS is measured by placing a fibre perpendicular to the LLS at different positions. The average photomultiplier anode voltage for the different fibre positions is measured to be 425 mV, and the maximum deviation of the photomultiplier anode voltage is 22% at a photomultiplier amplification voltage of 356 V. This LLS homogeneity is in accordance with Wurster et al., who measured a maximum deviation of 20 % [5].

**Figure 5:**
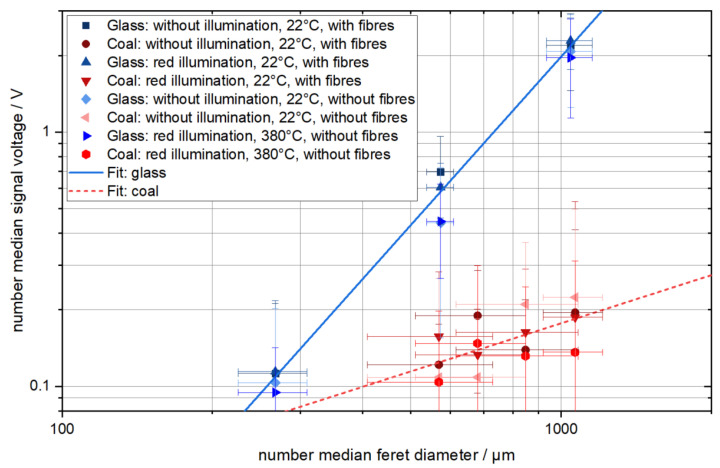
Number median signal voltage of the photomultiplier anode (signal voltage is the difference between peak voltage and average noise) gathered by measuring the light scattered on glass spheres or hard coal particles with different number median Feret diameter. Each point represents 20 measurements. The error bars represent the standard deviation.

The calibration for the dependence of the photomultiplier signal voltage (difference between peak voltage and average noise) on the particle size and material is shown in Figure 5. Each data point in the diagram represents 20 measurements.

Figure 5 shows that the photomultiplier signal of the glass spheres and hard coal particles of similar number median Feret diameter differ significantly. This is due to the different optical and geometrical properties of the particles. The illumination with red light and the presence of the fibre array in the measurement chamber does not systematically change the calibration curve. This is due to the optical filter, which hinders most of the red light from reaching the photomultiplier and due to the definition of the signal voltage as the difference between the peak voltage and the average noise, which cancels out differences caused by the presence of the fibre array. The calibration curves shown in Figure 5 at an increased temperature seem to be slightly lower than the other calibration curves. The hot air jet, which transports the test particles through the measurement chamber, may serve as a thermal gas lens because hot air has a lower refractive index than cold air [13], while the refractive index, and thus the reflectance, of glass increases with temperature [14] and therefore may cancel out the gas lens effect. The size reduction of the coal particles due to oxidation is negligible at these temperatures and residence times. However, the measurements at high temperature are within the standard deviation of the measurements at low temperature, and hence the temperature’s influence on the photomultiplier signal voltage is not significant.

The measurement results are fitted according to the following equation:

(1) $$\frac{U}{V}=A\cdot {\left(\frac{x}{\mu m}\right)}^{B}$$

where the photomultiplier anode number median signal voltage is U, the number of the median Feret diameter is x and the fit parameters are A and B. Equation (1) can serve as a calibration curve to determine the particle size to a corresponding photomultiplier signal. The fit parameters of the measurements shown in Figure 5 are summarized in Table 1.

The fit parameters in Table 1 show an almost quadratic dependence of the measurement signal on the diameter for the glass spheres. This is in accordance with theory because spherical particles have a projection area proportional to the square of their diameter, and the projection area is proportional to the intensity of scattered light if the particles are much larger than the wavelength of the used light [15,16]. The fit parameters for glass spheres show only a slight variation under the different measurement conditions mentioned in Table 1.

**Figure 6:**
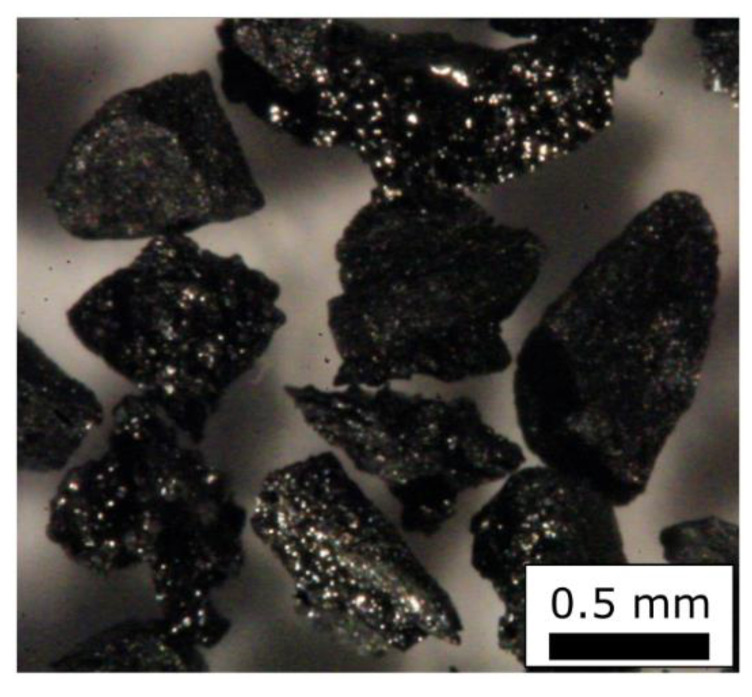
Coal particles applied in calibration (0.5 to 0.6 mm size fraction).

The coal particles show only a weak dependence of the photomultiplier anode number median signal voltage on the number of the median Feret diameter. The examination of a microscopic image of those particles provided in Figure 6 shows that those particles have a highly irregular shape and inhomogeneous light reflectance properties. Because it is not sure if the size, the shape or the inhomogeneous optical properties of those coal particles are the dominant factor for light scattering, those coal particles are not suited for calibration of measurement devices at all. However, the measurement results of these particles are used in the following paragraph to discuss the measurement of heterogeneous agglomerates containing soot and glass.

The size of the agglomerates containing both soot and glass spheres simultaneously is hard to determine because both components have different optical properties, and thus the calibration curves for both components are far apart from each other, as shown in Figure 5. Additionally, the weak dependence of the signal voltage on the diameter of the coal particles shows that the size of optically inhomogeneous objects is hard to determine using this measurement technique. The signal voltage of soot-glass agglomerates may strongly depend on the position of the glass spheres in the agglomerate, relative to the laser-light-sheet and the photomultiplier.

Because of these difficulties in size determination for soot-glass agglomerates, a different measurement procedure is necessary to investigate the detachment of agglomerates from single fibres. In this measurement procedure, the soot in the particle structures deposited on the fibres is oxidized at low flow velocities and thus without detachment. Afterwards the flow velocity is increased to observe the detachment of solely glass sphere agglomerates.

The device’s particle size measurement range is limited by the noise measured by the photomultiplier for small particles and the maximum voltage or the size of the LLS for large particles. The extrapolation of the calibration data results in a measurement range of 257 to 1523 µm for glass spheres and 403 to 15200 µm for coal particles. With this measurement range, neither the soot agglomerates (volume median diameter of 0.352 µm) nor the single glass spheres (volume median diameter of 6.8 µm), used to initially build the particle structures on the fibre, can be measured directly. This means that the particle structure on the fibre needs to be large enough, to allow the detachment of measurable agglomerates. The detachment of small particles is still possible also with large particle structures on the fibres, however, the large detached agglomerates contribute more to the change in the particle structure of the fibre (see Figure 1 for comparison) and are thus of primary interest of this project. 

The LLS-OPC improves detection of detached particle structures over pure visual observation of the deposit on the fibre by video recording and subsequent image analysis. By image analysis of the structures on the fibre array alone, it is difficult to determine if an agglomerate detached from the fibre or just rearranged. The LLS-OPC, in contrast, reliably detects and counts detached structures above the specified minimum size.

### 3.2. First Detachment Experiments

**Figure 7:**
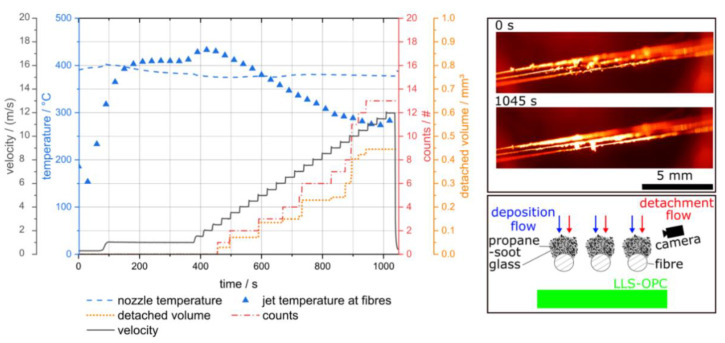
Detachment of soot–glass particle structures from 3 parallel fibres at high temperature and stepwise increasing flow velocity. The left side of the figure show the velocity and temperature conditions together with measurement data from the LLS-OPC, and the right side shows images taken by the camera.

Figure 7 shows a detachment experiment at elevated temperature.

The velocity in Figure 7 (black solid line) is the velocity calculated with the nozzle temperature (blue dashed line) at 1.013 bar (absolute) and the jet temperature at fibre array (blue triangles) is derived from calibration measurements, in which a thermocouple was placed at the position of the fibre array. The accumulated detached volume is calculated by the volume of glass spheres (equal to the step height in Figure 7) with a feret-diameter according to the measurement signal. With increasing flow velocity, the jet temperature at the fibre array first increases and then decreases because first some flow is needed in order of hot air reaching the fibre array and then further increase of the flow rate decrease the residence time of the air in the heaters and thus the air temperature. The first detached particle agglomerate was detected at a flow velocity of 2.5 m/s and its calculated volume is 0.03 mm^3^. This flow velocity is rather high, because typical filtration velocities in fibrous depth filters are commonly lower than 0.5 m/s in order to prevent particle bounce during deposition [17]. One aim of this research project is to investigate if significant detachment is possible at such low flow velocities, depending on the reactive-inert ratio, the size and the morphology of the particle structure and the detachment conditions used.

The images on the right side of Figure 7 show images of the three parallel fibres at the start (0 s) and at the end (1045 s) of the detachment experiment. The images show that the particle structure becomes smaller during the experiment and rearrangement in the flow direction. Thus, the rearrangement/ deformation of the particle structures can be observed with the novel apparatus. It is difficult to make a connection between the detected agglomerates and changes in the particle structure on the fibre because the different particle structures overlap and the particulate structures change on different parts of the fibre simultaneously. In addition, only a part of the deposit structure on the fibres is in focus, and the focus shifts along the fibre axis between the individual fibres due to the 45° arrangement of the fibre array with respect to the observation direction. 

Thus, only part of the deposit structure can be clearly observed. Here, another advantage of the LLS-OPC becomes apparent, as it captures the entire area of the deposits on the array and thus detects all detached agglomerates above a certain size limit, regardless of their spatial origin.

This experiment shows that detached particle agglomerates can be detected directly downstream of the fibre array using the LLS-OPC simultaneously with camera observation of the particle structures on the fibre array at high temperatures up to 430 °C. Therefore, this apparatus is suited to investigate the influence of the reaction, and thus the removal of particle structure components, on the detachment of large agglomerates from a single fibre or a fibre array under different conditions such as reactive content, particle size and temperature. A limitation of the current apparatus is that only glass sphere agglomerates with a light scattering equivalent diameter of single glass spheres larger than 257 µm can be detected, and thus the investigation of the detachment phenomena is limited to large agglomerates.

## 4. Conclusions

A novel apparatus was developed, which allows simultaneous measurement with a laser-light-sheet optical particle counter (LLS-OPC) and video recording in the same measurement chamber at high temperatures. This apparatus is used to oxidize soot in a particle structure deposited on single fibres and investigate the detachment of residual inert agglomerates from this particle structure due to gas flow. The performance of the new apparatus was investigated. The key findings are as follows:

A combination of the flow rectifier and the nozzle resulted in a plug-flow-shaped velocity profile. 

The temperature is lower in the centre of the air jet than at the boundaries because of former heating of the air stream from the outside. The temperature difference can be reduced by increasing the temperature of the first heater and decreasing the temperature of the second heater.

The red illumination for camera recording does not influence the photomultiplier signal of the LLS-OPC because the laser light has a wavelength of 532 nm (green) and can be separated from the red illumination using an optical filter and a suited photomultiplier.

The LLS-OPC calibration curves at a temperature of 470 °C show slightly lower signal intensities of the calibration particles than that at ambient temperature.

The determined measurement range of the LLS-OPC is 257 to 1523 µm for glass spheres and 403 to 15200 µm for coal particles.

The calibration curves of glass spheres and coal particles differ by almost one order of magnitude, hence the determination of the geometrical size of agglomerates containing soot and glass spheres simultaneously is difficult. Therefore, the soot is oxidized first and the flow velocity is increased second to detect agglomerates consisting solely of glass spheres.

The temperature of the gas jet, which flows out of the heated nozzle and impinges on the fibre array, shows a maximum in dependence of the gas flow rate.

Further measurements are planned to investigate the influence of different particle structure properties on the detachment of particle agglomerates from a cylindrical fibre in gas flow.

## Figures and Tables

**Table 1:** Fit Parameters for the Calibration Curves of the LLS-OPC.

Calibration particle	Fit Parameter A /-	Fit Parameter B /-
**glass:** complete data	4.78 × 10^−7^	2.199
**glass:** without illumination, 22 °C, with fibres	5.70 × 10^−7^	2.190
**glass:** red illumination, 22 °C, with fibres	5.22 × 10^−7^	2.200
**glass:** without illumination, 22 °C, without fibres	4.67 × 10^−7^	2.190
**glass:** red illumination, 380 °C, without fibres	3.75 × 10^−7^	2.218
**coal:** complete data	2.28 × 10^−3^	0.629
**coal:** without illumination, 22 °C, with fibres	5.48 × 10^−3^	0.506
**coal:** red illumination, 22 °C with fibres	1.44 × 10^−2^	0.361
**coal:** without illumination, 22 °C, without fibres	1.86 × 10^−5^	1.356
**coal:** red illumination, 380 °C without fibres	1.69 × 10^−2^	0.305

## Data Availability

Not applicable.
